# Implementing telemedicine with 5G technologies in a nursing home for reducing emergency admissions– study protocol of a mixed-methods study

**DOI:** 10.1186/s12913-024-11588-7

**Published:** 2024-09-23

**Authors:** Johanna Sophie Lubasch, Patrick Andreas Eder, Christian Kaiser, Andrea Diana Klausen, Daniel Overheu, Anja Partheymüller, Asarnusch Rashid, Simon Thomas Schäfer, Maximilian Scharonow, Insa Seeger

**Affiliations:** 1 Research Network Emergency and Intensive Care Medicine, School of Medicine and Health Sciences, Carl von Ossietzky Universität Oldenburg, Oldenburg, Germany; 2 Innovation Management, ZTM, Bad Kissingen, Germany; 3 Emergency Department, St. Josefs-Hospital Cloppenburg, Cloppenburg, Germany; 4 Evangelisches Krankenhaus Oldenburg, Oldenburg, Germany; 5 Clinic of Anesthesiology/Intensive Care Medicine/Emergency Medicine/Pain Therapy, Department of Human Medicine, School of Medicine and Health Sciences, University of Oldenburg, Klinikum Oldenburg AöR, Oldenburg, Oldenburg, Germany; 6 Anesthesia & intensive care medicine, St. Josefs-Hospital Cloppenburg, Cloppenburg, Germany

**Keywords:** Nursing Homes (MeSH Unique ID: D009735), Telemedicine (MeSH unique ID: D017216), Emergency Care Information Systems (MeSH Unique ID: D007256), Implementation, Organizational development, Avoiding hospital admission, Primary care, Ambulatory care

## Abstract

**Background:**

By transmitting various types of data, telemedical care enables the provision of care where physicians and patients are physically separated. In nursing homes, telemedicine has the potential to reduce hospital admissions in nonemergency situations. In this study, telemedicine devices were implemented with the new 5G mobile communications standard in selected wards of a large nursing home in Northwest Germany. The main aim of this study is to investigate which individual and organizational factors are associated with the use of telemedicine devices and how users perceive the feasibility and implementation of such devices. Moreover, it is investigated whether the telemedical devices help to reduce the number of emergency admissions.

**Methods:**

Telemedicine devices are implemented over an 18-month period using a private 5G network, and all users receive training. This study uses qualitative and quantitative methods: To assess the individual and organizational factors associated with the use of telemedicine devices, survey data from employees before and after the implementation of these devices are compared. To assess the perception of the implementation process as well as the feasibility and usability of the telemedical devices, the nursing staff, physicians, medical assistants and residents are interviewed individually. Moreover, every telemedicine consultation is evaluated with a short survey. To assess whether the number of emergency admissions decreased, data from one year before implementation and one year after implementation are compared. The data are provided by the integrated dispatch centre and emergency medical services (EMS) protocols. The interview data are analysed via structured qualitative content analysis according to Kuckartz. Survey data are analysed using multivariable regression analysis.

**Discussion:**

Learnings from the implementation process will be used to inform future projects implementing telemedicine in care organizations, making the final telemedicine implementation and care concept available to more nursing homes and hospitals. Moreover, the study results can be used to provide use cases for appropriate and targeted application of telemedicine in nursing homes and to define the role of 5G technologies in these use cases. If the intervention is proven successful, the results will be used to promote 5G network rollout.

**Trial registration:**

German Clinical Trials Register – trial registration number: DRKS00030598.

## Background

With increasing life expectancy, the need for acute medical care is increasing. This is indicated by the use of emergency departments (EDs) [1, 2] as well as emergency medical services [3]. Therefore, ED visits by nursing home residents play an important role [4], and a large proportion of patient transfers to hospitals can be avoided [5, 6]. Often, nursing home residents are transported to the ED solely for liability reasons [7]. In many cases, ED visits lead to hospitalization, which in turn puts patients at risk for cognitive and functional decline, infections, and falls [8, 9]. Possible solutions to reduce hospital admissions for these patients have emerged, such as the implementation of community emergency paramedics [10] and the construction of networks with general practitioners (GPs) or emergency physicians (EPs).

Another approach to reduce avoidable hospital admissions among nursing home residents is the provision of telemedical support [11]. Telemedicine can be used for remote patient monitoring to share patient medical information, such as laboratory results, with a physician at another location or to provide communication between physicians, health care professionals and patients despite spatial distance [12]. In the context of residential care, GPs and emergency physicians can regularly monitor health status via ward televisits in routine situations or remotely assess the situation via teleconsils in subacute situations and evaluate whether hospitalization is necessary. Since telemedicine is dependent on the transfer of data (e.g., video calls, the sharing of clinical data), the 5th generation wireless system (5G) offers new opportunities for telemedicine since it has good performance in terms of high speed, low latency, and high bandwidth [13].

Telemedicine provided to nursing homes was found to have the potential to reduce transport and (emergency) admissions to the hospital [14–17]. Although telemedicine cannot entirely replace face-to-face visits, it has the potential to reduce the burden and workload of visits in nursing homes for physicians and to save nursing staff from unnecessary distraction during routine tasks [18]. Complementing in-person visits with telemedicine consultations in nursing homes has the potential to address the difficulties arising from physician shortages in rural areas of Germany [18]. However, additional research is needed on the implementation of telemedical devices in nursing homes [18, 19]. Although there is initial evidence that families of nursing home patients are interested in telemedicine [20], further research should specifically focus on residents’ and families’ opinions of telemedicine and telehealth use in nursing homes [15].

The need to embed telemedical care into existing structures without creating additional organizational and administrative work in the daily care routine is one challenge when implementing telemedical devices in nursing homes [18]. Moreover, it is important that telemedicine devices are accepted by all stakeholders involved [20, 21]. To address these challenges, telemedicine applications should be adapted to meet the needs and competencies of not only users but also residents [19, 20].

In addition, the implementation of interventions in care settings should always consider that care settings are complex organizations [22]. According to Braithwaite et al. [22], complexity is characterized by the fact that different organizations respond differently to the same input, and organizational behaviors cannot be predicted by extrapolating from the past. On the other hand, Braithwaite et al. [22] describe that organizations exhibit inertia regarding targeted changes in, for example, behavioral patterns or organizational cultures. As a result, complex systems, such as nursing homes, cannot be changed solely in a targeted manner based on initiatives by individuals [22]. To address these circumstances during implementation, basic principles of organizational development should be applied, such as adapting the intervention to the specific organization and involving organizational members in the change process in a participatory manner [22, 23].

## Method/design

### Aim of the study

The aim of this study is to implement and evaluate telemedical devices using 5G technologies in selected nursing home wards in a large nursing home in Northwest Germany. The use of telemedical devices aims to reduce EMS missions in nonemergency situations and therefore to reduce avoidable hospitalization for residents and to relieve EMS.

### Setting

The study is conducted in a nursing home in rural Northwest Germany. The charitable nursing home offers approximately 300 inpatient care places at two sites, with the main site having ten different wards. The 3-year study is conducted from January 2022 through December 2024.

### Study design

The selected wards of the nursing home are each equipped with telemedical devices, and the nursing staff is trained to use the devices. Implementation is accompanied by an outcome and process evaluation because when evaluating complex interventions, not only the effect on outcomes but also the process of implementation must be examined to identify mechanisms of action and contextual factors [24, 25].

The implementation process as well as the corresponding facilitating and hindering factors of the implementation are examined based on the consolidated framework of implementation science (CFIR) [24, 25]. Therefore, how and whether the telemedical devices are used and accepted, whether the target groups can be reached, which mechanisms of action can be identified, and how the users of the telemedical devices as well as the residents of the nursing home perceive the consultation with the telemedical devices are assessed. The results are used at the end of the implementation process to make any necessary changes in the implementation. Data is conducted via employee surveys, short-evaluations of each telemedicine consultation and qualitative individual interviews with the nursing staff and residents of the nursing home, general practitioners, specialists, medical assistants (MFAs) and physicians of the on-call service.

**Fig. 1:**
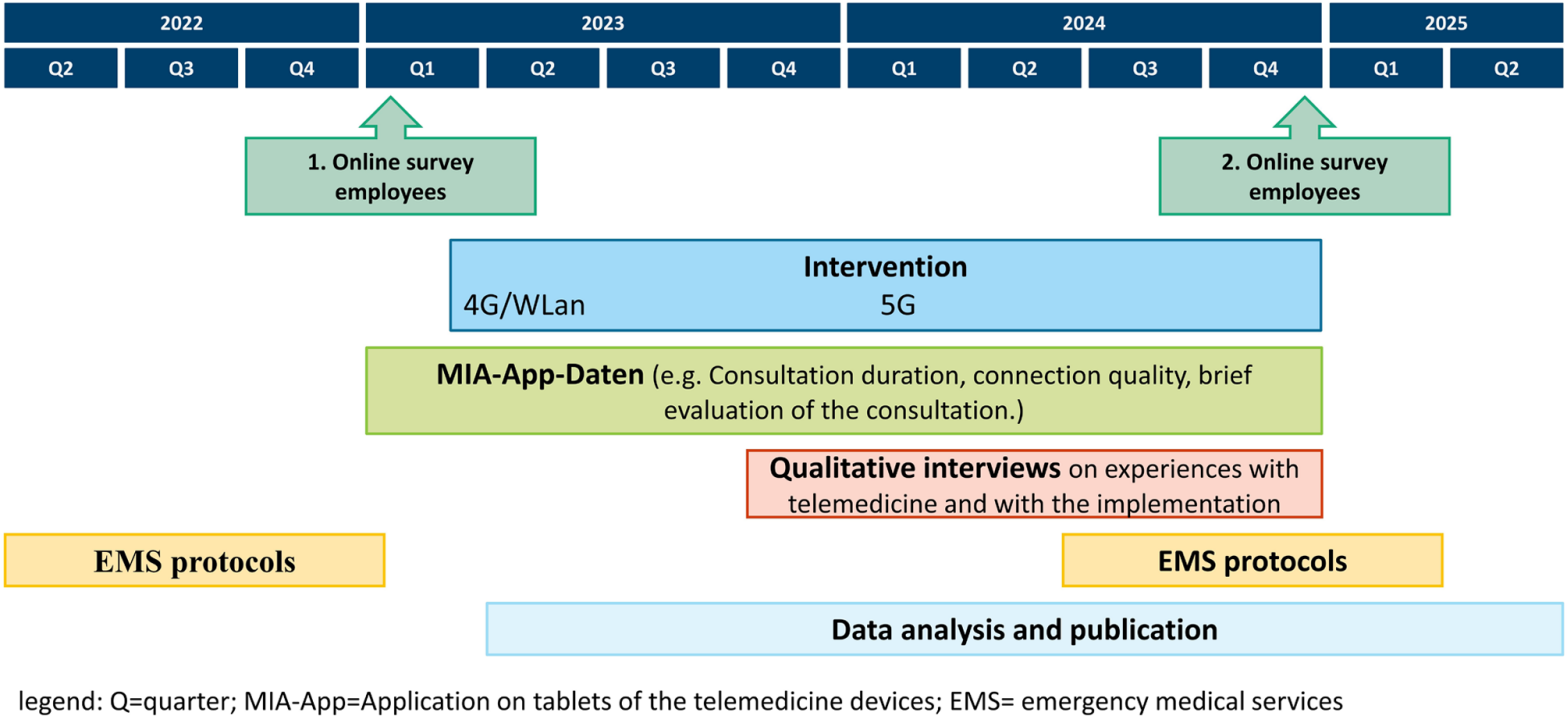
Study design

To assess whether the intervention reduced EMS missions, EMS missions are monitored over a period of one year before the start and at the end of the implementation period (until December 2024) (see Fig. 1). The employee survey is conducted before (June-August 2023) and at the end of the implementation period (October-November 2024). The individual qualitative interviews are conducted approximately 9 months after the start of the implementation period (August-October 2024). Short-term evaluations of telemedicine consultations take place during the entire implementation period.

### Principles of the intervention

Within the present study, telemedical devices are provided in selected wards of a large nursing home in Northwest Germany. The devices are either provided in backpacks or on ward trolleys, depending on the preferences of the practicing nurses. The backpacks and ward trolleys contain common examination instruments (e.g., blood pressure cuffs, stethoscopes, and blood glucose meters), which are used by certified nursing staff and transmit the patient’s vital signs via Bluetooth to the attending physician using an enclosed tablet (Samsung Galaxy Tab S7 5G, Samsung Electronics Co., Ltd., Korea). The attending physicians (general practitioners, specialists in private practice and hospital physicians) are equipped with the necessary terminal devices and trained. Vital signs and video chats are managed on the tablet via the MIA app (for more information, see https://www.ztm.de/mia). MIA is a certified class I medical device according to the Medical Device Law Implementation Act, the “Medizinprodukterecht-Durchführungsgesetz” (MPDG). According to the MIA data protection concept, the medical facility is responsible for the lawful collection of data with the MIA app. It decides which measurements are to be performed on the residents and obtains the declaration of consent from the residents in advance. To ensure that telemedical devices can also be used during night services, when participating wards may also be cared for by nursing staff from nonparticipating wards, all nursing staff of the nursing home are trained. Together with the nurses from the nursing home and physicians providing the telemedicine consultation, use cases for which the telemedicine devices can be used effectively and sensibly are identified. To effectively implement telemedicine devices and adapt them to the needs of the nursing home according to aspects of organizational development [22, 26], the results of the first cross-sectional survey are used to address possible factors hindering the intention to use telemedicine devices. The devices are implemented stepwise over the course of 18 months. In the first step, devices are implemented that can be used with long-term evolution (LTE) standards. This includes all examination instruments.

**Fig. 2:**
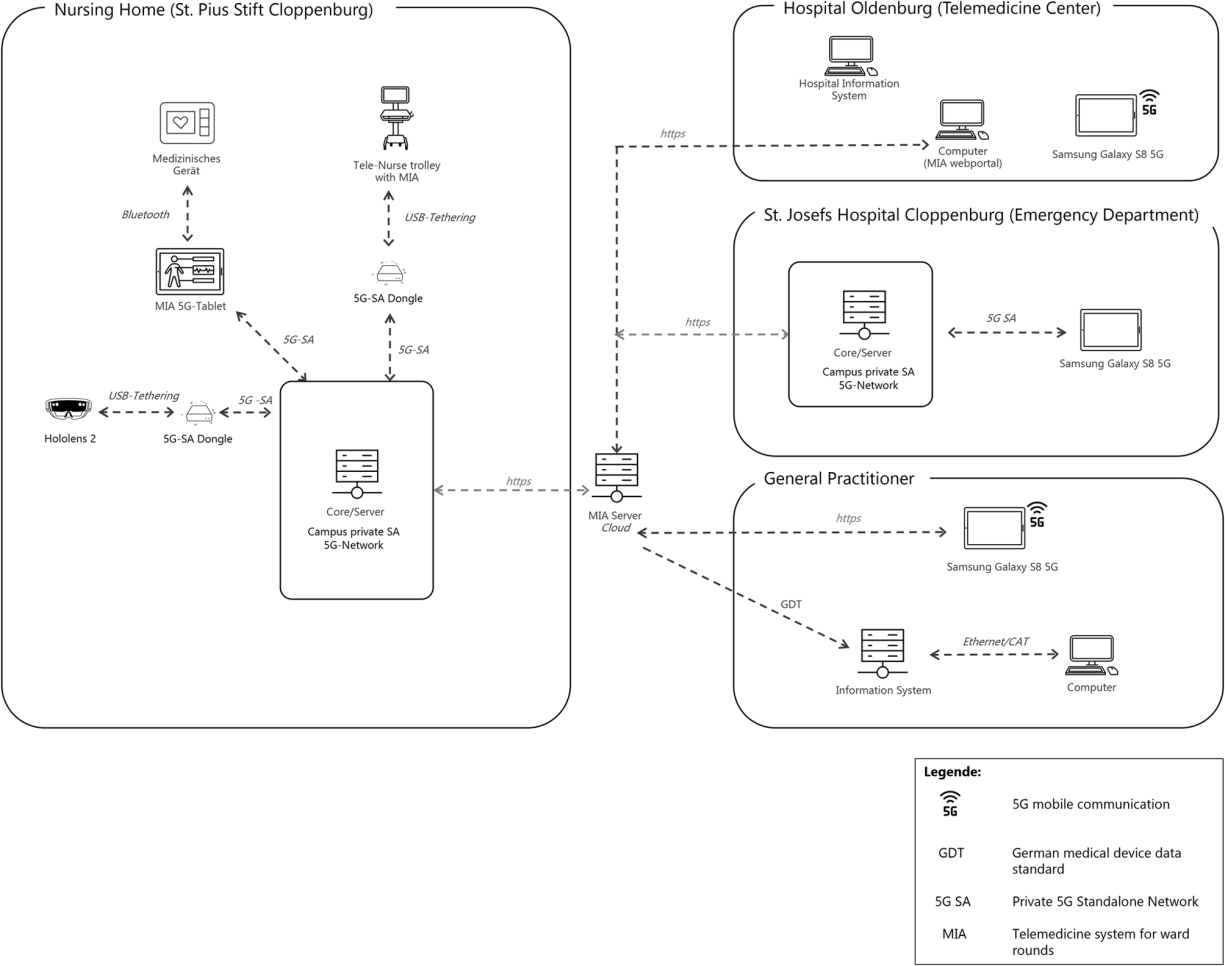
Global architecture of the SERNV-project

We use a private 5G network (3GPP compliant 5G-Core as a Service; 5GaaS) as a standalone dedicated network for private use (CampusGenius GmbH, Dresden/Berlin, Germany) installed and commissioned on site at the nursing and medical facility (Fig. 2). 5G antennas are installed and operated directly in the ceilings of the wards. In the second step, the 5G network is implemented in one ward to enable high-resolution and low-latency video telephony, the transmission of live ultrasound images and the use of augmented reality (AR) data glasses (e.g., Microsoft HoloLens 2; Microsoft Corp., USA). AR can offer an environment where users can move around, interact with, and manipulate 3D virtual objects and see the results of their actions in which objects are integrated into a 3D real environment in real time [27]. In this use case, an interactive whiteboard is augmented on which both the doctor and the carer can exchange information about the patient (functions: writing, taking photos, 3D drawing in the room on the patient, screen sharing). As HoloLens 2 itself is not equipped with a 5G-capable router, a 5G dongle was connected via a USB tethering function. Therefore, one participating ward of the nursing home and the ED of one hospital conducting the telemedicine consultations are equipped with a 5G network. Telemedicine consultations in other wards and/or hospitals participating in the study will be carried out via Wi-Fi.

### Logic model of the study

**Fig. 3:**
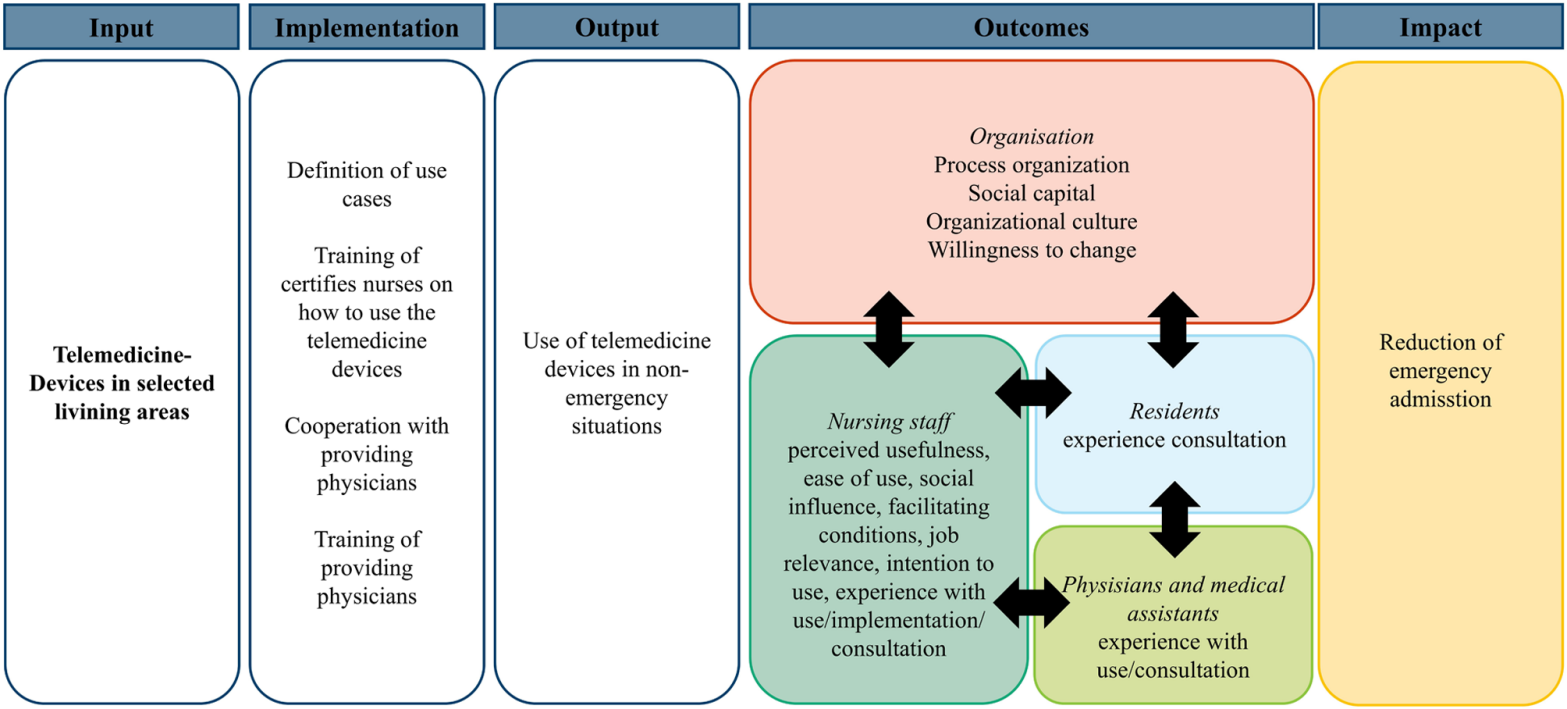
Logic model of the intervention

The logic model (Fig. 3) that guides the evaluation of the implementation of telemedical devices is developed on the basis of the Medical Research Council Framework for the evaluation of complex interventions [24]. Individual and organizational factors associated with the use of telemedical devices are measured from the perspectives of the nursing staff. Data on the implementation process are collected from the perspectives of the nursing staff, residents, physicians and medical assistants. The number of emergency admissions as the impact of the intervention is assessed by data from the emergency dispatch centre and EMS protocols.

The selection of individual and organizational factors associated with the use of telemedical devices is based on the unified theory of acceptance and use of technology (UTAUT) according to 28 [28], supplemented by the relevance for professional activity from the extended technology acceptance model (TAM2, precursor of UTAUT) [29], technology readiness and organizational factors. Technology readiness is included in the survey because it extends the UTAUT through personality influences [30]. Since the organizational context should always be considered when implementing technology in care facilities, process organization, social capital, and readiness for change are also examined as organizational factors [24, 25] (Fig. 3).

### Evaluation

#### Sample

All employees with nursing duties are included in the survey. Thus, approximately 350 employees can be included. Based on previous experience, a participation and response rate of 25% is assumed. To obtain a comprehensive picture of employees’ attitudes towards telemedicine, all employees in nursing homes who have routine contact with patients are included. These are the following professional groups: certified nursing staff, nursing assistants, caregivers and housekeeping staff. Participation is only possible for employees who have sufficient knowledge of German to be able to read and complete the consent form and questionnaire. For the qualitative interviews, a heterogeneous group of care professionals are recruited (e.g., with regard to the ward they are working in, age, professional experience) so that as many different perspectives as possible can be recorded. With respect to the GPs and specialists in private practice and the physicians in the on-call service, all the participating physicians participated in the interviews if possible. The residents of the nursing home are invited to participate in the interviews if they are capable of providing consent and have participated in a telemedical consultation at least once. The qualitative individual interviews are conducted with 8 employees (*n* = 5 general practitioners and specialists in private practice, *n* = 5 medical assistants, *n* = 5 physicians of the on-call service and *n* = 8 residents of the nursing home).

#### Recruitment

Employees are recruited via an information event planned by management at the beginning of the project. Following the event, employees have the opportunity to participate in the survey via a QR code on their personal devices. Employees who are unable to attend the event also are informed about the study at the night watch meeting and given the opportunity to participate in the survey. Nursing home staff and residents are recruited for qualitative interviews via notices and/or personal approaches. Residents are included only if they are able to consent independently. Since the general practitioners and specialists in private practice and the physicians of the on-call service also attend project meetings as project partners, recruitment is carried out by addressing them at the project meetings.

#### Measures

##### Intention to use

Intention to use is assessed with 3 items from the German version of the UTAUT [28, 31] (Cronbach’s α 0.971). The item is rated on a 7-point Likert scale (from strongly agree to strongly disagree).

##### Perceived usefulness

Perceived usefulness is assessed with 4 items from the German version of the UTAUT [28, 31] (Cronbach’s α 0.951). The items are rated on a 7-point Likert scale (from strongly agree to strongly disagree). Perceived usefulness is defined as the extent to which an individual believes that using the system helps them improve their job performance [28].

##### Perceived ease of use

Perceived ease of use is assessed with 4 items from the German version of the UTAUT [28, 31] (Cronbach’s α 0.922). The items are rated on a 7-point Likert scale (from strongly agree to strongly disagree). Perceived ease of use is assessed because the less effort required to adopt an innovation, the more its use can increase job performance [32].

##### Subjective norms

Subjective norms were assessed with 3 items from the German version of the UTAUT [28, 31] (Cronbach’s α 0.948). Subjective norms are defined as “a person’s perception that most people who matter to him or her think that he or she should or should not perform the behaviour in question” and is thus a direct determinant of behavioural intention [33]. The items are rated on a 7-point Likert scale (from strongly agree to strongly disagree).

##### Facilitating conditions

Facilitating conditions are assessed with 4 items from the German version of the UTAUT [28, 31] (Cronbach’s α 0.733). Facilitating conditions are defined as the extent to which an individual believes that organizational and technical infrastructure is in place to support the use of the system [28]. The items are rated on a 7-point Likert scale (from strongly agree to strongly disagree).

##### Job relevance

Job relevance is measured by 2 items of the TAM2 [29] (Cronbach’s α 0.80–0.95). Job relevance measures perceptions regarding the degree to which the telemedicine backpack is applicable to one’s work [29]. The items are rated on a 7-point Likert scale (from strongly agree - strongly disagree).

##### Use experience

The experience of using a technical system is a moderator of the relationships between perceived usefulness, perceived ease of use, subjective norms, facilitating conditions and intention to use [28]. The experience of use is subsequently assessed after each use of the telemedicine backpack or terminal device with the providing physicians in the MIA app using a short questionnaire. In addition, qualitative interviews are used to gain a deeper understanding of the usage experience.

##### Process organization

With 6 items, process organization records how the effectiveness of organizational processes in the nursing home is perceived (e.g., waiting times for medical care, coordination between nursing home wards). The scale was developed as part of the Cologne Patient Questionnaire [34] and adapted for the staff context [35] (Cronbach’s α 0.86). Because the scale was developed in the hospital context, it is adapted to the nursing home context in the present study. In addition, qualitative interviews are used to gain a deeper understanding of changes in organizational processes.

##### Social capital

Social capital is measured using the validated 6-item SCAPO-E scale by 41 [36] (Cronbach’s α 0.93). Social capital is defined as the characteristics of social organizations, such as networks, norms, and social trust, that facilitate coordination and cooperation in an organization for mutual benefit [37].

##### Change attitude

Change attitude is measured using the German validated version of the 6-item Change Attitude Scale [38, 39]. A change attitude is defined as “[.] a psychological state in which members of an organization are committed to implementing an organizational change and have confidence in their collective abilities to do so” [40]. A change attitude has been identified in the literature as an important factor for successful implementation of innovation in organizations [40–42].

##### Monitoring

According to the CFIR, monitoring data are used for process evaluation [24]. Therefore, consultation data (e.g., consultation duration, examination instruments, brief evaluation of the consultation) are collected for each telehealth consultation via the MIA app. The brief evaluation is assessed using items from the Telehealth Usability Questionnaire (TUQ) [43].

Quality assurance during study execution is safeguarded by the standards of questionnaire development [44, 45] and pretesting [46] and by creating the survey using the online survey service SoSci-Survey.

#### Individual Interviews

For formative evaluation, guideline-based individual interviews were conducted. The interviews were led by one researcher according to a semistructured interview guideline and last up to 90 min [47, 48]. The interview guidelines were developed following the existing standards, study objectives and research questions of the study. Topics are experiences with telemedical devices, as well as the implementation of the devices and changes in organisational processes.

##### Data from the emergency dispatch center and EMS protocols

EMS operations in the nursing home are recorded using digital routine data from the emergency dispatch center and EMS protocols (e.g., age, sex, duration of operation, destination (hospital or outpatient, on-site), type of ambulance) (see Table 1).

**Table 1:** Data collection and analysis methods for the summative and formative evaluations

	Data collection methods	Timepoints	Content	Measurement instruments	Data analysis
**Implementation process**	*Survey of healthcare professionals* *n* = approx. 350 employees; assumed participation and response rate of 25%	T0 – before implementation T1 – after implementation	*Primary endpoint*: Intention to use telemedicine device *Secondary endpoint*: UTAUT-model components, organisational aspects	German version of the UTAUT [28, 31]	1. Descriptive statistics 2. Comparison between T0 and T1 data 3. Multivariable regression analysis, controlled for possible confounders
	*Short-evaluation of consultation*: *n* = approx. 30 consultations evaluated by nurses and physicians	In the course of the intervention	Use experience Routine data of consultation (e.g. duration, examination instruments used)	Telehealth Usability Questionnaire (TUQ) [43]	1. Descriptive statistics 2. Comparison of data collected during intervention with 4G and 5G network
	*Qualitative interviews with nurses*,* residents*,* physicians and medical assistants* Interviews with *n* = 8 employees, *n* = 5 general practitioners and specialists in private practice, *n* = 5 medical assistants, *n* = 5 physicians of the on-call service and *n* = 8 residents	In the course of the implementation	Experiences with telemedical devices and the implementation; potential changes in the process organization; need for improvement of the intervention	Development of a semistructured guideline based on the research question, a literature review and standards for the preparation of interview guidelines [47, 48]	Transcription, qualitative structured content analysis [49, 50] to break down the complexity of the material and identify categories
**Reduction of EMS missions**	*Data from emergency dispatch centre and EMS protocols*	One year before the intervention One year at the end of the intervention	*Primary endpoint*: Number of emergency admissions *Secondary endpoint*: transfer to hospital vs. home treatment	Routine data	1. Descriptive statistics

### Data management and monitoring

The online survey service SoSci-Survey is used to create the survey. The raw datasets are created directly from the completed online questionnaires. This means that the data do not need to be entered manually. The data is analysed using analysis software (e.g., SPSS and Mplus). The individual interviews are recorded using a digital audio recorder and transcribed according to established standards [50]. The digitally recorded focus group interviews will be transcribed verbatim. The content is analysed using MAXQDA software. The online survey and interview data will be processed and stored on the university’s IT services systems. The data of the MIA app is initially stored on servers of the administration of the district of Cloppenburg in Northwestern Germany. The data is transferred from the administration to the University of Oldenburg via a password-protected cloud.

#### Data analysis

##### Employee survey

Survey data from healthcare professionals prior to the implementation of the intervention are compared with data collected after the intervention using multivariable regression analysis, controlling for possible confounders (e.g., ward, work experience). Since the individual survey participants before versus after the intervention are expected to differ to some extent (due to fluctuation, rotation and nonresponders), the data are primarily treated as cross-sectional rather than longitudinal within the analysis. All derived effects are calculated with 95% confidence intervals and corrected for multiple testing. To handle missing data, multiple imputation is conducted.

##### Interview data

The interview data are analysed via structured qualitative content analysis according to Kuckartz [49]. Therefore, audio recordings are first transcribed verbatim and pseudonymized according to transcription standards [50]. First, the main categories derived a priori from the interview guidelines are developed deductively. Transcripts are then coded, and subcategories are inductively formed during the coding process. The computer-aided coding of text segments into categories is performed using the program MAXQDA Analytics Pro (Version 2020, VERBI GmbH, Berlin, Germany). The entire coding process is conducted by two independent coders, followed by discussions to establish a consensus.

##### Data from the emergency dispatch centre and EMS protocols

For the summative evaluation, data from the emergency dispatch centre and EMS protocols prior to the intervention are compared with data after the intervention via multivariable regression analysis. Possible confounders (e.g., age, sex) are controlled for within the analyses.

## Discussion

Within the SERNV study, telemedical devices are implemented with a private 5G network in selected nursing home wards of a large nursing home in Northwest Germany. Therefore, the intervention aims to relieve the employees of the nursing home and the EMS in nonemergency situations. However, the focus is always on ensuring adequate care for residents. Telemedicine devices are therefore only used when transport to a hospital is not necessary, and adequate care can be provided in the nursing home by certified nursing staff.

A strength of the study is that it uses a mix of quantitative and qualitative methods to assess the implementation process of a complex intervention in a real-life setting. Moreover, the use cases in which telemedical devices are used are defined together with nurses and physicians to tailor the intervention to the specific needs of the nursing home. As a limitation, the study is only conducted in one nursing home due to limited resources, which prevented us from controlling for any organization-specific factors during the analysis. Moreover, the collection of survey and interview data might be affected by low response and participation rates, which could lead to selection biases. To reduce this risk, active recruitment strategies, including repeated reminders, are conducted. Moreover, the questionnaire is kept as short as possible. Further adverse events that could have a negative impact on the progress of the project could occur in the form of a lack of 5G network rollout and a lack of acceptance of the telemedicine devices. In the event of a lack of network rollout, the aim is to implement telemedicine devices under current network conditions, if possible. The lack of acceptance of telemedicine devices is addressed by measures of organisational development. Furthermore, the dynamic development of the COVID-19 pandemic could affect outcomes as well as the feasibility of the SERNV project. If undesirable events occur, the research team plans to have constructive dialogues with those responsible at the nursing home to find solutions that will enable the project to continue as planned.

Learnings from the implementation process will be used to inform future projects implementing telemedicine in care organizations, making the final telemedicine implementation and care concept available to more nursing homes and hospitals. Moreover, the results of this study can be used to provide use cases for appropriate and targeted application of telemedicine in nursing homes and to define the role of the 5G network in these use cases. If the intervention is proven successful, the results will be used to promote 5G network rollout.

### Dissemination plan

The study results are summarized in the final report. Moreover, the results are disseminated in the national and international scientific community via publications and conference presentations. If the intervention is successful, the aim should be to expand the 5G network and to ensure the widespread use of telemedical devices in nursing homes.

### Patient and public involvement

The project does not include any genuine patient or public involvement components. However, employees in nursing homes are closely involved in the process of selecting telemedical devices. Moreover, the experiences of nurses, residents, physicians and medical assistants are constantly collected during the implementation process and used to adapt the intervention to the needs of the respective persons.

## Data Availability

The datasets used and/or analysed during the current study are available from the corresponding author upon reasonable request.
